# Alcohol consumption: An important epidemiological factor in COVID-19?

**DOI:** 10.7189/jogh.10.020335

**Published:** 2020-12

**Authors:** Abhimanyu Vasudeva, Tejas K Patel

**Affiliations:** 1 Department of Physical Medicine and Rehabilitation, All India Institute of Medical Sciences, Gorakhpur Gorakhpur, Uttar Pradesh, India; 2 Department of Pharmacology, All India Institute of Medical Sciences, Gorakhpur, Gorakhpur, Uttar Pradesh, India

The COVID-19 pandemic has caused a massive loss of human life in addition to severe economic consequences. Evidence suggests that SARS-Cov-2 gains entry into the human body mainly through the respiratory system, although other routes are also being studied. The spectrum of symptoms may range from mild to severe manifestations such as hypoxia with acute respiratory distress syndrome (ARDS). The progression to ARDS may occur in a matter of days. [1]. Recent meta-analyses on COVID-19 estimate the percentage of patients who developed ARDS as follows: 9.4% (95% confidence interval (CI) = 5.6%-13.2%) [2], 15.7% (95% CI = 5.0%–30.4%) [3], 19.5% (95% CI = 5.0%-40.3%) [4] and 32.8% (95% CI = 13.7%-51.8%) [5]. Damage to the alveolar epithelial cells is considered the main cause of COVID-19-related ARDS [6]. Various biomarkers and cytokines such as the angiotensin converting enzyme 2, interleukin-10, tumour necrosis factor, and vascular endothelial growth factor are linked to severe ARDS [7].

Alcohol gets absorbed via the bronchial circulation directly across the ciliated epithelium of the conducting airways. Vaporization during exhalation is followed by condensation as the air cools the vapors in the trachea eventually leading to a higher concentration of alcohol [8] which modifies airway-epithelium host defenses by altering cytokine release, barrier function, and ciliary function [9]. Alcohol also causes oxidative stress and alterations in alveolar macrophage functions [10]. This alteration in pulmonary defense could enhance the risk of acquiring SARS-Cov-2 infections. Moreover, ARDS is more likely in patients who have an injury to the lung by pathogens including viruses. A recent meta-analysis of 13 observational studies found an association between alcohol consumption and the risk of developing ARDS (1.89; 95% CI = 1.45-2.48) among adults [11]. There appears to be a higher risk of acquiring COVID-19 related ARDS in chronic alcoholics (**Figure 1**). The World Health Organization (WHO) in its public information factsheet of COVID-19 states that heavy alcohol use increases the risk of ARDS, which is one of the complications of COVID-19. This factsheet was to caution the public regarding alcohol consumption and the fact that it is neither protective nor preventive against COVID-19, a common myth due to its important role in alcohol-based sanitizers for hand hygiene [12]. Alcohol consumption has been somewhat omitted from the perspective of risk factors for the severity of COVID-19 disease.

**Figure 1:**
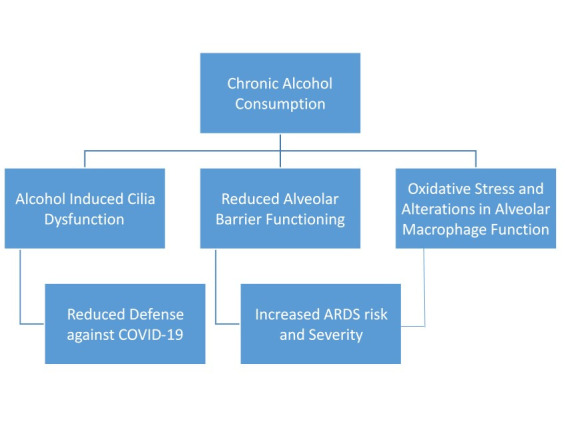
Deleterious effects of alcohol consumption on lung and its possible implications on COVID-19.

This hypothesis has profound implications as alcohol consumption is widespread throughout the world. Moreover, alcohol is hardly recognized as injurious to lung health as opposed to other organs such as the liver by the public at large. COVID-19 is an illness without a medication or a vaccine as of date, it would be in the public interest to highlight this association as a preventive measure. This virus may stay with us for a long time if not forever and changing drinking habits may go a long way in both reducing the spread of infection as well as reducing the chances of persons developing a serious illness. Conditions such as old age, hypertension, and diabetes, to name a few, have been identified as factors that can increase the risk of mortality due to this dreaded virus.

**Figure:**
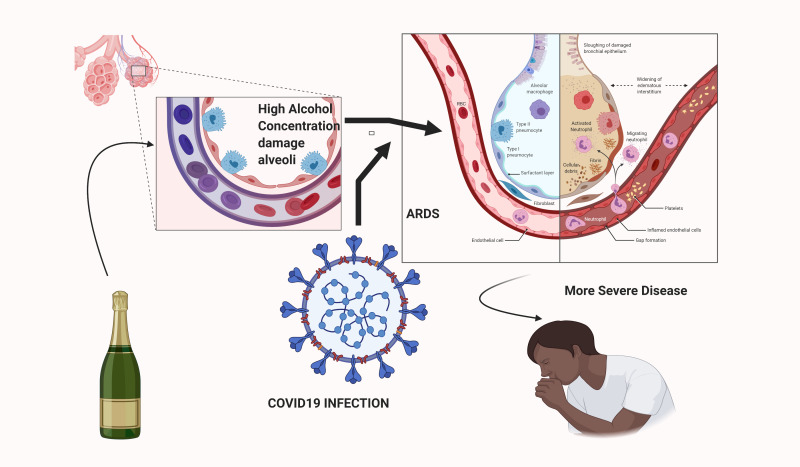
Photo: Created by Dr Samantak Sahu, Postgraduate trainee (MD), Department of Physical Medicine and Rehabilitation, All India Institute of Medical Sciences, New Delhi (used with permission).

## CONCLUSION

We hypothesize that consumption of alcohol would lead to an increased risk of developing SARS-Cov-2 infections as well as severe illness.
